# Preparation of Mucoadhesive Oral Patches Containing Tetracycline Hydrochloride and Carvacrol for Treatment of Local Mouth Bacterial Infections and Candidiasis

**DOI:** 10.3797/scipharm.1004-18

**Published:** 2010-12-14

**Authors:** Rana M. Obaidat, Ammar Bader, Wafa Al-Rajab, Ghassan Abu Sheikha, Aiman A. Obaidat

**Affiliations:** 1 Faculty of Pharmacy, Jordan University of Science and Technology, Irbid, Jordan; 2 Faculty of Pharmacy, Al-Zaytoonah Private University of Jordan, Amman, Jordan

**Keywords:** Ethyl cellulose, Carbopol 934, Mucoadhesion, Oral infections

## Abstract

The specific aim of this work was to prepare mucoadhesive patches containing tetracycline hydrochloride and carvacrol in an attempt to develop a novel oral drug delivery system for the treatment of mouth infections. The bilayered patches were prepared using ethyl cellulose as a backing layer and carbopol 934 as a matrix mucoadhesive layer. Patches were prepared with different loading amounts of tetracycline hydrochloride and carvacrol. The antimicrobial activity was assessed for the prepared patches using the disc-diffusion method against the yeast *Candida albicans* and five bacterial strains, including *Pseudomonas aeruginosa*, *Escherichia coli*, *Bacillus cereus*, *Staphylococcus aureus*, and *Bacillus bronchispti*. In this work, we highlighted the possibility of occurrence of a synergistic action between carvacrol and tetracycline. The best formulation was selected based on microbiological tests, drug release, *ex-vivo* mucoadhesive performance, and swelling index. Physical characteristics of the selected formulations were determined. These included pH, patch thickness, weight uniformity, content uniformity, folding endurance, and patch stability.

## Introduction

Oral diseases are a health problem in immuno-suppressed patients around the world since the oral cavity provides a diverse environment for colonization by a wide variety of microorganisms [1–6]. Most of mouth infections are mainly due to candidiasis and bacterial infections. It has been reported that more than 300 bacterial species have been identified in the periodontal pockets [6].

Tetracyclines are a group of broad-spectrum antibiotics, which were introduced into clinical practice in the late 1940s [7–11]. They are bacteriostatic antibiotics which interfere with the bacterial protein synthesis. A further mechanism may be associated with the ability of tetracyclines to scavenge reactive oxygen radicals produced by neutrophils which will prevent further tissue destruction. Thus, tetracyclines may have general antiproteolytic properties. It is now well established that tetracyclines have anticollagenase properties unrelated to their antibacterial activity [11]. Tetracycline HCl has a broad spectrum activity inhibiting both Gram negative and Gram positive organisms, including the beta-lactamase-producing strains against which penicillin is ineffective. Tetracycline HCl has been shown to be effective against many of the common periodontopathic bacteria, in particular, *Prevotella intermedia* and *Porphyromonas gingivalis* [10].

One of the major concerns about antibiotic usage, particularly in long-term low dosage regimes, is that bacteria may develop resistance to the antibiotic. Microorganisms can develop resistance after exposure to sub-inhibitory concentrations of antibiotics [12]. Recently, a combination of two or more antibiotics has been recognized as an important method for, at least, delaying the emergence of bacterial resistance [11]. Besides, antibiotic combinations may also produce desirable synergistic effects in the treatment of infections [11, 12].

Essential oils, such as oregano oil, have been shown to be useful as antimicrobial and antifungal agents; competing pharmaceutical antibiotics such as streptomycin and penicillin and antifungal agents such as nystatin and amphotericin have been proved to be effective in their ability to eliminate microbes [12]. All this has been accomplished without promoting the development of drug-resistant strains and other problems often attributed to the use of standard antibiotics. Carvacrol, 5-isopropyl-2-methylphenol, has been identified as the chief constituent of oregano oil’s extraordinary properties. It is generally recognized as a food additive and a flavoring agent [12, 13]. Essential oils containing carvacrol are biostatic and biocidal against many bacterial strains, yeasts, and fungi in laboratory media and have consequently attracted considerable research attention as potential food preservatives. Carvacrol has also been shown to inactivate microorganisms in biofilms or stainless steel surfaces [13]. It has anti-fungal activity against *Candida* species. The biocidal mode of action of carvacrol on bacteria is similar to that of other phenolic compounds where it increases the microbial cell membrane permeability to protons and potassium ions. This will induce cell membrane damage. In addition, carvacrol was also shown to rapidly desensitize pain receptors [12].

The consequent need for local drug delivery has been recognized since many years. To date, a great number of local drug delivery systems and devices have been proposed for oral and dental applications, including fibers, strips, films, gels, sponges, microparticles, etc [12–19]. It is speculated that a higher mucoadhesive strength of the delivery system will lead to prolonged retention of the device in the oral cavity and increased absorption across mucosal tissues [20–24]. Most of the previously formulated drug delivery systems involved treatment of bacterial infections alone or candidiasis.

The main objective of this study was to develop an oral mucoadhesive controlled-release delivery system containing tetracycline HCl and carvacrol. This system is intended for local treatment of both oral candidiasis and bacterial infections. Selection of tetracycline and carvacrol as active ingredients in the proposed oral patches was based on the expected complementary action from both of them.

## Experimental

### Materials

Tetracycline HCl was kindly provided by Dar Al-Dawaa Company [Jordan]. Carbopol 934 Q.C no. 1001333 and glycerol were obtained from Scharlau Chemie [Spain]. Carvacrol and ethyl cellulose CAS number 9004-57-3 were obtained from Sigma Chemical Co. [USA]. The water used throughout all the experiments was HPLC grade and was obtained from Acros Organics [Belgium]. All reagents were of pharmaceutical grade and used as supplied without further treatment.

### Microorganisms

Microorganisms were obtained from Dar Al-Dawaa Company [Jordan]. Two strains of gram-negative bacteria *Escherichia coli* [ATCC 8739] and *Pseudomonas aeruginosa* [ATCC 9027]; three strains of gram-positive bacteria *Staphylococcus aureus* [ATCC 6538], *Bacillus cereus* [ATCC 14579], and *Bacillus bronchispti* [ATCC 4617]; and yeast *Candida albicans* [ATCC 10231] were used. The cultures of the bacteria were maintained in their appropriate agar plates at 4°C throughout the study.

### Preparation of the bilayered mucoadhesive patches

Bilaminated films were produced by a casting/solvent evaporation technique using different combinations of polymers and drugs. The backing membrane was prepared by dissolving ethyl cellulose [5%] in chloroform with 1.35 g of propylene glycol [30% w/w of polymer content] as a plasticizer. The plasticized ethyl cellulose solution was poured into a 10 cm^2^ glass mould on a leveled surface and the solvent was allowed to evaporate at ambient temperature.

The mucoadhesive layer was prepared using carbopol 934 as the polymer-forming matrix. Two grams of carbopol 934 were soaked in 70 ml water for 24 h, after which 30 ml of ethanol was added; glycerol at a concentration of 25% w/w of polymer content was added as a plasticizer. The dispersion was stirred at 150 rpm using a mechanical stirrer for 3 h. Various loading amounts of tetracycline HCl and carvacrol were added to 20 ml of the polymer dispersion to obtain various formulations as shown in Table 1. The pH of the resultant dispersion was measured, and it had an average value of 6.5±0.6. The plasticized polymeric solution was poured into a glass mould containing the backing membrane and oven-dried at 30 °C for 96 h. The dried films were peeled from the glass mould and cut into circular shapes of small size. They were kept at 25 °C in a desiccator containing a saturated solution of sodium dichromate [Na_2_Cr_2_O_7_], which provided an environmental condition of 55% relative humidity [RH] for at least 2 days before testing. Patches with any imperfections, entrapped air, or mean thickness variations of ± 5% were excluded from further analysis and use.

### Antimicrobial activity

Screening of the prepared patches for antimicrobial activity was performed according to the disc diffusion method [24, 25]. It was performed using an 18-h culture at 37 °C in 10 ml of Mueller-Hinton broth [Oxoid, England] with sterile saline solution. The disc diffusion test was performed on Mueller-Hinton agar. Plates were cultured with appropriate test organisms overnight. For *C. albicans*, Sabouraud dextrose agar and Sabouraud dextrose broth were used. On the following day, three to five freshly grown colonies of bacterial strains were inoculated in 20 ml of Mueller-Hinton medium in a shaking water bath for 4–6 h until a turbidity of 0.5 McFarland 1 × 10^8^ colony forming unit [CFU/ml] was reached. Final inocula were adjusted to 5 × 10^5^ CFU/ml. The inoculum [200 μl] from the final inocula was seeded onto the agar plate and uniformly spread with a sterilized cotton spreader over the surface. Discs of diameter 5 mm from each prepared films were attached to the product and deposited on Mueller-Hinton agar plates, except for *C. albicans* which was deposited on Sabouraud dextrose agar plates. The plates [prepared in triplicate for each strain] were then incubated at 37 °C for 24 h and the inhibition zones were measured after 24 h for bacteria and 48 h for the fungus *C. albicans* [26]. Standard 5-mm tetracycline antibiotic discs [30 μg of tetracycline HCl/disc] were used as positive controls. Each film was cut into discs of similar sizes. Discs [5 mm] from films without active ingredients were also used as negative control.

### In vitro drug release

*In vitro* release of tetracycline HCl from the matrices through dialysis membrane was investigated using Franz-type diffusion cells with an effective diffusion area of 2.3 cm^2^. A quantity of 15 ml of pH 7 phosphate buffer solution maintained at 37 °C and stirred at 100 rpm was used as the receptor medium. The matrices [donor phase] were placed in contact with the dialysis membrane such that the backing layer faced the donor compartment and the adhesive film faced the receiver compartment fastened with an O-ring. At predetermined time intervals, 3-ml samples of the receiving phase were withdrawn for analysis and replaced with an equal volume of fresh buffer to maintain sink conditions. The amount of tetracycline HCl and carvacrol released was determined at 275 nm and 283 nm using a UV spectrophotometer [Cavy 1E Varian, USA]. Method of analysis was spectra-first derivative method [27]. The method was validated and each release test was performed for a minimum of three times. UV-scan of placebo solution showed no absorbance at the analytical wavelength.

### Ex-vivo mucoadhesive force

The mucoadhesive strength was determined by measuring the force of detachment or the force of adhesion and the *ex-vivo* adhesion time. These are the most frequently studied parameters for determination of adhesive properties.

**Fig. 1.**
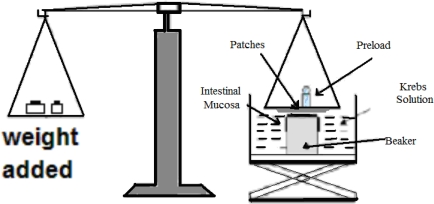
Schematic of the two-arm balance used in the assessment of the bioadhesion of the films

The two-arm balance method reported by Parodi [26] with minor modifications was used to assess the bioadhesiveness of the films. Fresh rabbit buccal mucosa section [2×2 cm and 2 mm thick] was fixed at the bottom of a smaller beaker placed in a larger beaker [Figure 1]. Krebs solution was added to the larger beaker up to the upper surface of the mucosa. The patch was attached to the upper clamp and the platform was slowly raised until the patch surface came in contact with mucosa. Two minutes of contact time was found to give optimum mucoadhesive strength. Further increase in contact time did not affect the mucoadhesive strength, whereas decreased contact time resulted in less mucoadhesive strength resulting from insufficient time for entanglement of polymer chains with mucin. After a preload time of 5 minutes, a weight [g] was added to the second arm of the balance until the film was detached from the buccal mucosa. The weight [g] required for the detachment was recorded. The method was validated and the average values were reported after triplicate experiments.

### Measurement of mucoadhesion time

The mucoadhesive performance of the buccal patches was evaluated using rabbit buccal mucosa tissue [2×2 cm and 2 mm thick]. The time taken for the film to detach from the mucosal section in a well-stirred beaker was used to assess the mucoadhesive performance. The fresh cut tissue was fixed to the side of the beaker with glue. Before addition of the buffer, the films were attached to buccal mucosal tissue by applying light force [approximately 0.5 N] for 20 sec. The beaker was then filled with 800 ml phosphate buffer and kept at a temperature of 37 °C. A stirring rate of 150 rpm was maintained to simulate the buccal and saliva movement. The time for the film to detach from the mucosal tissue was recorded up to 12 h. The average values were reported after repeating the experiments three times.

### Percent swelling

Patches were weighed individually [designated as W1] and placed separately in a test tube filled with simulated saliva [2.38 g Na2HPO4, 0.19 g KH2PO4, and 8 g NaCl per liter of distilled water adjusted with phosphoric acid to pH 6.8] incubated at 37 ± 0.5 °C and examined for any physical changes. After 2 h, patches were removed from the test tube and excess surface water was removed carefully using filter paper. The swollen patches were then reweighed [W2] and the swelling index [%] calculated using the following equation:

Eq. 1. $$\text{Swelling}\;\text{Index}[\%]=\frac{\left(w2-w1\right)}{w1}\times 100$$

The experiments are run in triplicates.

### Physical characteristics of the patches

#### Folding endurance

The folding endurance of the patches was determined by repeatedly folding a patch at the same place until it broke or was folded up to 250 times without breaking.

#### Surface pH of the films

For determination of the surface pH, three films from each patch were allowed to swell by keeping them in contact with 1 ml of distilled water for 2 h at room temperature. The pH was recorded by placing the electrode in contact with the surface of the patch and allowing it to equilibrate for 1 minute.

#### Content uniformity, thickness, weight uniformity, and stability

Drug content uniformity was determined by dissolving each patch in 30 ml of ethyl alcohol at 37 °C temperature and filtering through a 0.45 μm membrane filter [Millipore, USA]. The filtrate was evaporated and the residue was dissolved in 100 ml of phosphate buffer [pH 6.8]. After 24 h, 5 ml solution was withdrawn and diluted with phosphate buffer [pH 6.8] up to 20 ml, filtered through a 0.45 μm membrane filter and analyzed at 275 and 283 nm for tetracycline and carvacrol content.

The thickness of the selected patches was measured using a screw gauge. The weight variation of the patches was measured by weighing random pieces of patches of identical size. Thickness and weight variation measurement were obtained from six different areas in the patches including the edges and the middle parts.

Stability testing of the prepared patches was performed by keeping the patches in glass Petri dishes lined with aluminum foil and stored in desiccators at 25 °C temperature and 55% RH for 6 months. Changes in appearance and drug content of the stored patches were investigated at the end of the storage period.

Readings from all the previous experiments were performed in triplicate and the average values were reported.

## Results and Discussion

### Antimicrobial activity

Table 2 summarizes the antimicrobial activity of the patches against *Candida albicans*, *Staphylococcus aureus, Pseudomonas aeruginosa*, *Escherichia coli*, *Bacillus cereus*, and *Bacillus bronchispti.* The antimicrobial activity was assessed using the disc diffusion method and the mean inhibition zone diameters [MIDs] were recorded. Comparison was made with standard tetracycline disc and the effective MID was considered to be higher than 15 mm. The standard tetracycline disc showed activity against *Staphylococcus aureus* [MID = 30 mm], *Bacillus cereus* [MID = 20 mm], *Bacillus bronchispti* [MID = 15 mm], and *Escherichia coli* [MID = 15 mm].

The results showed that discs containing carvacrol alone [F2–F7] showed excellent activity against *Candida albicans* in amounts ≥40 μg/disc [F3], while discs containing tetracycline alone [F8–F13] did not show any activity against this microorganism [MID = zero]. However, either tetracycline alone or carvacrol alone was effective against *Staphylococcus aureus* and both of them did not show any activity against *Pseudomonas aeruginosa* [MID < 10 mm]. Tetracycline alone was very effective against *Bacillus cereus and Bacillus bronchispti* [MID values ranges from 15 to 20 mm] while carvacrol alone was not effective [MID < 12 mm]. Formulations containing tetracycline were all effective against *Escherichia Coli* [MID = 15 mm] while carvacrol was effective in amounts ≥85 μg/disc.

Results for formulations containing tetracycline and carvacrol [F14–F19] showed that they were all effective against all tested microbes. Activity of these formulations against *Pseudomonas aeruginosa* was excellent and MID values ranged from 15 to 20 mm. These results indicate a synergistic action between tetracycline and carvacrol since both of them were separately ineffective against *Pseudomonas aeruginosa*. Also, their inhibition efficiency increased [MID > 30 mm] against *Bacillus cereus* when they were used in combination which also indicates a synergistic effect.

It is expected that the synergistic effect is due to enhancement of the permeability of tetracycline through the bacterial cell wall. This is due to the fact that one of the proposed mechanisms for the development of bacterial resistance against tetracycline might be due to decreased antibiotic influx through the bacterial cell wall [10]. On the basis of these results, the best formulations were those containing both tetracycline and carvacrol in combination [F14–F19].

### In vitro drug release

**Fig. 2.**
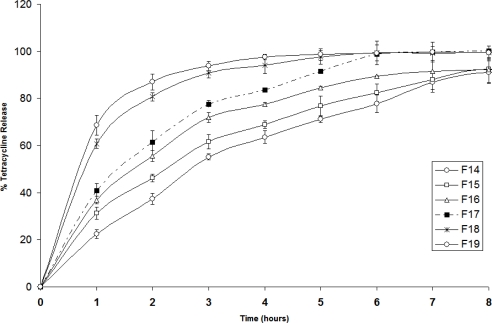
Release profiles of tetracycline HCl from patches F14–F19 in Franz diffusion cell at 37 °C and pH 7 [n =3].

Figure 2 illustrates the percent tetracycline release profiles from the various patches [F14–F19]. These patches contain various combinations of tetracycline and a constant amount of carvacrol. It can be clearly seen that in all patches the percent released decreased with time. This could be due to the extensive swelling of the polymers creating a thick gel barrier, thus making drug diffusion to be slower with time. The slowest release is observed in the formulation containing the lowest concentration of tetracycline [F14]. Patches with higher loading doses of tetracycline showed fast release; however, patches with lower loading doses resulted in a sustained near zero-order release. This could be explained due to high water solubility of tetracycline HCl, which increased the swelling of the polymer and facilitated drug diffusion. As shown in Figure 2, patches F14–F16 demonstrated sustained release of tetracycline for more than 6 h.

**Fig. 3.**
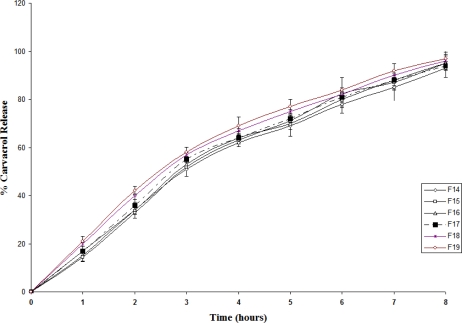
Release profiles of carvacrol from patches F14–F19 in Franz diffusion cell at 37 °C and pH 7 [n =3].

The release profile for carvacrol is shown in Figure 3. All the three formulas showed sustained release effect for carvacrol. It appears that there is no significant difference between the release profile for the various formulas; although increase in tetracycline concentration caused a slight increase in the release rate as can be observed in F19.

The release mechanism of the drug from the patches was investigated using the Korsemeyer-Peppas equation [29]

Eq. 2. $$\frac{M_{t}}{M_{\propto }}=k\cdot t^{n}$$

where *M_t_/M_∞_* is the fractional release of the drug; *t* denotes the release time; *K* is a constant incorporating structural and geometric characteristics of the controlled release device; and *n* is the release exponent, indicative of the drug release mechanism. A linear form of this equation is

Eq. 3. $$\text{Log}\left(\frac{M_{t}}{M_{\propto }}\right)=\text{Log}(k)+n\;\text{Log}(t)$$

Kinetic parameters are obtained from a plot of Log [*M*_t_/*M_∞_*] versus Log [*t*], where *n* represents the slope and log [*K*] represents the intercept. In systems which could swell, factors affecting release kinetics are liquid diffusion rate and polymeric chain relaxation rate. When the liquid diffusion rate is slower than the relaxation rate of the polymeric chains, the diffusion is Fickian and *n* = 0.45. If 0.45 < *n* < 0.89; it indicates anomalous or non-Fickian mechanism. This indicates that liquid diffusion rate and polymer relaxation rate are of the same order of magnitude. Whereas when the relaxation process is very slow compared with the diffusion, case II transport occurs and n ≥ 0.89. This indicates that the release mechanism involves polymer erosion.

Kinetic parameters for both tetracycline HCl and carvacrol obtained for patches F14–F19 according to this equation are presented in Table 3. The diffusion exponent [*n*] for tetracycline HCl ranged from 0.50 to 0.75, while for carvacrol it ranged from 0.72 to 0.85. The correlation coefficient (r^2^) was in the range of 0.97–0.99 for various formulations. This is an indication of anomalous, or non-Fickian, release mechanism.

### Ex-vivo mucoadhesive force

**Fig. 4.**
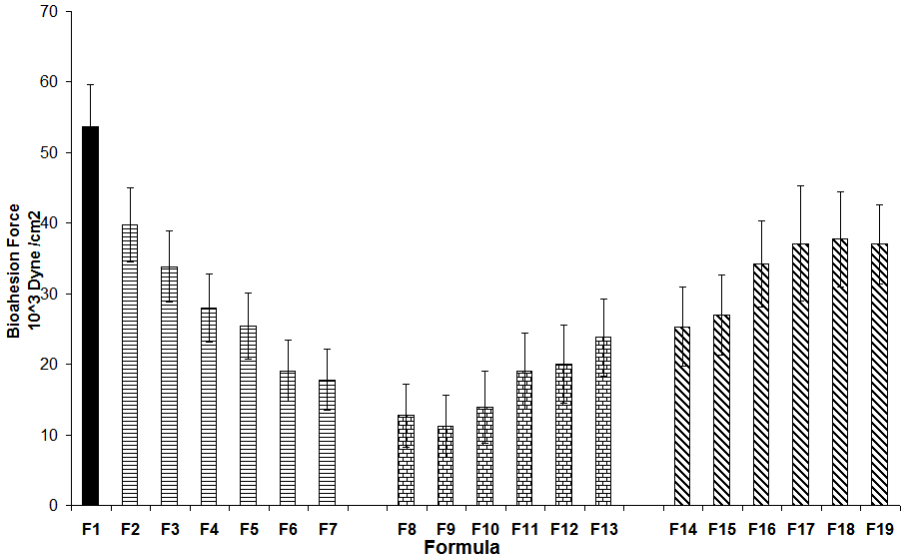
Ex-vivo mucoadhesion force of the patches. The average values are presented ± STD.

Figure 4 shows the ex-vivo mucoadhesion force for the various patches prepared. The maximum bioadhesion force was observed for carbopol 934 [F1]. A possibility of hydrogen bonding between carboxylic groups of the polymer with the sugar residues in oligosaccharide chains in the mucus membranes, resulting in the formation of a strengthened network between the polymer and mucus membranes. Earlier work with carbopol 934 polymer has clearly indicated that the presence of carboxyl groups (58%–68%) in its structure contributes to its strong bioadhesion [16].

Addition of carvacrol alone to the carbopol 934 polymer [F2–F7]; resulted in reduced bioadhesion. This could be because of chemical interaction between carvacrol and carbopol 934. This in turn decreases the possibility of interaction between the polymer and the sugar residues in oligosaccharides chains in the mucus membranes and further decreasing polymer mucoadhesion. Figure 4 also illustrates that tetracycline addition [F8–F13] to the carbopol 934 polymer resulted in significant reduction in bioadhesion. This could be explained by the formation of hydrogen bonding between tetracycline and the polymer. In the rest of the formulations [F14–F19], an increase in the bioadhesion strength in the patches containing both tetracycline HCl and carvacrol was noted. This could be explained by possibility of hydrogen and π-π bonding between the phenolic hydroxyl groups of carvacrol with tetracycline, thus resulting in the polymer’s availability for interaction and adhesion to the mucus membranes. A previous study proved that addition of drugs that could form hydrogen bonds with the hydrophilic groups of the polymer is expected to decrease the bioadhesive force of the polymer [28]. According to this study, addition of a third component able to form hydrogen bonds with the drug is expected to free the bioadhesive polymeric chains and thereby enabling the polymer to retain its bioadhesive properties with the mucus membranes. This needs further study.

The bioadhesive strength exhibited by the films was satisfactory in maintaining them in the oral cavity. This aspect was further confirmed by measurement of mucoadhesive time. During mucoadhesive time studies, none of the patches was dropped from the buccal tissue within 12 h of experiment time.

### Water uptake [swelling index]

Hydration is required for a mucoadhesive polymer to expand and create a proper macromolecular mesh of sufficient size and also to induce mobility in the polymer chains in order to enhance the interpenetration process between the polymer and mucin. Polymer swelling permits a mechanical entanglement by exposing the bioadhesive sites for hydrogen bonding and/or electrostatic interaction between the polymer and the mucus network. However, a critical degree of hydration of the mucoadhesive polymer exists where optimum swelling and bioadhesion occurs.

**Fig. 5.**
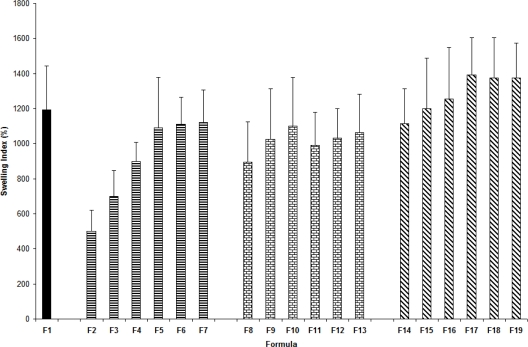
Swelling indexes of the patches. The average values are presented ± STD.

Figure 5 summarizes the swelling index values for the patches. Swelling behavior of carbopol 934 is attributed to the uncharged COOH group that is hydrated by forming hydrogen bonds with water, thus extending the polymer chains. Carbopol 934 polymers exhibit very good water sorption properties where they swell in water up to 1000 times their original volume and 10 times their original diameter to form a gel when exposed to a pH environment above 4 to 6. Because the pKa of these polymers is 6.0 to 6.5, the carboxylate moiety on the polymer backbone ionizes, resulting in repulsion between the native charges, which adds to the swelling of the polymer. Presence of carvacrol alone in low amounts [F2–F4] caused a decrease in the swelling index. On the other hand, carvacrol in higher amounts [F5–F7], tetracycline [F8–F13], and combination of both [F14–F19] did not cause any detectable effect on swelling index for the patches. The patches did not show any appreciable changes in their shape and size during the 3-h soaking in simulated salivary solution.

### Physical characteristics of the patches

The physical characteristics of selected patches are summarized in Table 3. The folding endurance was measured manually by folding the film repeatedly at a point until it broke. The breaking time was considered as the end point. The folding endurance test did not develop any visible cracks or breaks [>250 times], thus showing good film elasticity.

Considering the fact that acidic or alkaline pH may cause irritation to the buccal mucosa and influence the degree of hydration of the polymers, the surface pH of the buccal films was determined to optimize both drug permeation and mucoadhesion. The surface pH of the patches was determined in order to investigate the possibility of any side effects in the oral cavity. As shown in Table 3, the average pH value is 6.31 ± 0.51 for various patches. This shows that there is no significant difference between the pH of the patches and the mouth pH [6.68 ± 0.55] [30].

Drug content in the formulations was uniform and the standard deviation did not exceed 0.7% of the theoretical concentration. This indicates that the drug was dispersed uniformly throughout the films. The average thickness of the patches was 0.15 ± 0.05 mm. Weight variation was uniform in the patches with an average value of 0.05 ± 0.01 g.

Physical appearance was good for all the prepared patches. Stability of the patches was tested over 6 months. All the prepared patches were stable in physical appearance, uniformity of weight, and thickness. The films did not exhibit any color changes during the storage period. Results showed that the decrease in drug content did not exceed 2%. This suggests that the stability was satisfactory for the drug and the patches during the storage period.

## Conclusions

A novel mucoadhesive bilayered film consisting of ethyl cellulose as a backing layer and carbopol 934 as a matrix-forming layer was prepared using the casting method. Combination of tetracycline and carvacrol showed excellent activity against *Candida albicans* and the selected bacterial strains. Evidence of synergism between the two components against *Pseudomonas aeruginosa* and *Bacillus cereus* was shown and it needs further investigation. The prepared patches showed a sustained release action for more than 6 h, acceptable bioadhesion, and stability. The best suggested patch according to our results was F14 [0.01 g tetracycline and 1.1 mg carvacrol per 10 cm^2^ of film] which contained the least effective concentrations of the two active components and the maximum delayed release and an acceptable mucoadhesion force.

## Figures and Tables

**Tab. 1.** Tetracycline HCl and carvacrol contents of the prepared patches

**Formula**	**Tetracycline HCl [g/10 cm^2^]**	**Carvacrol [mg/10 cm^2^]**
**F1**	0	0
**F2**	0	0.50
**F3**	0	0.80
**F4**	0	1.10
**F5**	0	1.40
**F6**	0	1.70
**F7**	0	2.00
**F8**	0.01	0
**F9**	0.02	0
**F10**	0.03	0
**F11**	0.04	0
**F12**	0.05	0
**F13**	0.06	0
**F14**	0.01	1.10
**F15**	0.02	1.10
**F16**	0.03	1.10
**F17**	0.04	1.10
**F18**	0.05	1.10
**F19**	0.06	1.10

**Tab. 2.** Mean inhibition zone diameters of the prepared patches obtained on colonies of the microorganisms under study.

**Formula**	**Mean inhibition zone diameter (MID) [mm]**					
	***Candida albicans***	***Staph. aureus***	***Pseud. aeruginosa***	***Bacillus*** **C*ereus***	***Bacillus Bronchis.***	***Escher. Coli***
**Std. tetracycline disc**	0	30	0	20	15	15

**F1**	0	0	0	0	0	0
**F2**	<10	0	<10	<10	<10	<10
**F3**	20	15	<10	<10	<10	<10
**F4**	>30	15	<10	<10	<10	<10
**F5**	>30	15	<10	<10	<10	<10
**F6**	>30	>30	<10	12	<10	15
**F7**	>30	>30	<10	12	<10	15
**F8**	0	>30	0	15	<15	<15
**F9**	0	>30	0	15	15	15
**F10**	0	>30	0	20	15	15
**F11**	0	>30	0	20	20	15
**F12**	0	>30	0	20	20	15
**F13**	0	>30	0	20	20	15
**F14**	>30	>30	15	>30	>30	23
**F15**	>30	>30	15	>30	>30	25
**F16**	>30	>30	20	>30	>30	25
**F17**	>30	>30	20	>30	>30	25
**F18**	>30	>30	20	>30	>30	25
**F19**	>30	>30	25	>30	>30	25

**Tab. 3.** Summary of the physical characteristics of patches F14–F19.

**Patch**	**Release kinetic parameters**		**pH [±STD]**	**Content uniformity [%] [±STD]**	**Thickness [mm] [±STD]**	**Weight variation [mg] [±STD]**
	**Tetracycline HCl**	**Carvacrol**				
**F14**	n = 0.75	n = 0.85	6.4 [±0.3]	98% [±0.6]	0.16 [±0.02]	0.05 [±0.01]
	K= 0.29	K= 0.76				
	R^2^ = 0.99	R^2^ = 0.97				
**F15**	n = 0.74	n = 0.84	6.3 [±0.4]	99% [±0.5]	0.15 [±0.02]	0.05 [±0.01]
	K = 0.21	K= 0.78				
	R^2^ = 0.99	R^2^ = 0.97				
**F16**	n = 0.54	n = 0.82	6.5 [±0.3]	98% [±0.7]	0.15 [±0.01]	0.05 [±0.01]
	K = 0.30	K= 0.72				
	R^2^ = 0.99	R^2^ = 0.98				
**F17**	n = 0.51	n = 0.81	6.1 [±0.7]	100% [±0.9]	0.15 [±0.01]	0.04 [±0.01]
	K = 0.33	K= 0.71				
	R^2^ = 0.99	R^2^ = 0.97				
**F18**	n = 0.51	n = 0.74	6.3 [±0.5]	99% [±0.8]	0.15 [±0.01]	0.05 [±0.01]
	K = 0.37	K= 0.65				
	R^2^ = 0.99	R^2^ = 0.98				
**F19**	n = 0.50	n = 0.72	6.3 [±0.5]	99% [±0.7]	0.16 [±0.01]	0.05 [±0.01]
	K = 0.41	K= 0.63				
	R^2^ = 0.99	R^2^ = 0.98				
